# The efficacy and safety of pre-hospital plasma in patients at risk for hemorrhagic shock: an updated systematic review and meta-analysis of randomized controlled trials

**DOI:** 10.1007/s00068-024-02461-7

**Published:** 2024-02-17

**Authors:** Mohamed Abuelazm, Hazem Rezq, Abdelrahman Mahmoud, Mohammad Tanashat, Abdelrahman Salah, Othman Saleh, Samah Morsi, Basel Abdelazeem

**Affiliations:** 1 Faculty of Medicine, Tanta University, Tanta, Egypt; 2 Faculty of Medicine, Al-Azhar University, Cairo, Egypt; 3 Faculty of Medicine, Minia University, Minia, Egypt; 4 Faculty of Medicine, Yarmouk University, Irbid, Jordan; 5 Faculty of Medicine, Zagazig University, Zagazig, Egypt; 6 Faculty of Medicine, The Hashemite University, Zarqa, Jordan; 7 Department of Radiation Oncology, UT Texas MD Anderson, Houston, TX USA; 8 Department of Cardiology, West Virginia University, Morgantown, WV USA

**Keywords:** Plasma, Trauma, Shock, Bleeding, Systematic review, Meta-analysis

## Abstract

**Background and objective:**

Plasma is a critical element in hemostatic resuscitation post-injury, and its prompt administration within the prehospital setting may reduce the complications resulting from hemorrhage and shock. Our objective is to assess the efficacy and safety of prehospital plasma infusion in patients susceptible to hemorrhagic shock.

**Methods:**

We conducted our study by aggregating randomized controlled trials (RCTs) sourced from PubMed, EMBASE, Scopus, Web of Science, and Cochrane CENTRAL up to January 29, 2023. Quality assessment was implemented using the Cochrane RoB 2 tool. Our study protocol is registered in PROSPERO under ID: CRD42023397325.

**Results:**

Three RCTs with 760 individuals were included. There was no difference between plasma infusion and standard care groups in 24-h mortality (*P* = 0.11), 30-day mortality (*P* = 0.12), and multiple organ failure incidences (*P* = 0.20).

Plasma infusion was significantly better in the total 24-h volume of PRBC units (*P* = 0.03) and INR on arrival (*P* = 0.009). For all other secondary outcomes evaluated (total 24-h volume of packed FFP units, total 24-h volume of platelets units, massive transfusion, vasopressor need during the first 24 h, any adverse event, acute lung injury, transfusion reaction, and sepsis), no significant differences were observed between the two groups.

**Conclusion:**

Plasma infusion in trauma patients at risk of hemorrhagic shock does not significantly affect mortality or the incidence of multiple organ failure. However, it may lead to reduced packed red blood cell transfusions and increased INR at hospital arrival.

**Supplementary Information:**

The online version contains supplementary material available at 10.1007/s00068-024-02461-7.

## Introduction

Hemorrhagic shock, classified as a form of hypovolemic shock, leads to insufficient oxygen delivery to the tissues. When left untreated, hemorrhagic shock inevitably results in the patient’s demise, accounting for over 60,000 fatalities annually in the USA and approximately 1.9 million deaths worldwide [1]. Current management plans aim to prevent coagulopathy by decreasing the use of crystalloids and increasing blood component infusions such as plasma, platelets, and packed red blood cells (PRBCs), which are given in equal ratios [2]. The approach mentioned above is referred to as “damage-control resuscitation”; its early initiation has been shown to enhance hemostasis, increase survival rates, and reduce complications related to hemorrhage [3].

Plasma use was first proposed in late 1970 in the USA; it was theorized that it would lessen coagulopathy, coagulopathy-associated acidosis, and hypothermia (termed lethal triad), which typically lead to uncontrolled bleeding [4]. Plasma has been proven to repair the injured endothelium; improve the inflammatory response; and reduce endothelial cell permeability, hyperfibrinolysis, and the risk of coagulopathy-associated acidosis [5–8]. COMBAT trial suggested that using blood products is beneficial with longer transport time; however, it also indicated that prehospital administration of plasma did not affect survival [9]. In addition to that, the PAMPer trial suggested that prehospital plasma administration to patients at risk of hemorrhagic shock reduced the 30-day mortality rate and the median prothrombin-time ratio compared to standard care [10]. Nevertheless, evidence regarding prehospital plasma infusion for hemorrhagic shock is still lacking [11].

With the recent publication of the PREHO-PLYO trial [12], our goal is to provide an updated synthesis of evidence regarding the effectiveness and safety of prehospital plasma infusion for enhancing outcomes in patients susceptible to hemorrhagic shock.

## Methodology

### Protocol registration

This systematic review and meta-analysis strictly adhered to the Preferred Reporting Items for Systematic Reviews and Meta-Analyses (PRISMA) guidelines [13] and the Cochrane Handbook of Systematic Reviews and Meta-analysis [14]. Moreover, this review’s protocol was prospectively published and registered in PROSPERO with ID: CRD42023397325.

### Data sources and search strategy

Two reviewers (B.A. and M.A.) systematically conducted electronic database searches, including PubMed, EMBASE, Web of Science, Scopus, and Cochrane CENTRAL, until January 29, 2023. We manually searched PubMed for any recent RCT other than the studies included before the submission, and there were none. Detailed search terms and results can be found in [SI].

### Eligibility criteria

We included randomized controlled trials (RCTs) meeting specific criteria defined by PICO:

- Population (P): Trauma patients at risk of hemorrhagic shock, defined by systolic blood pressure (SBP) ≤ 70 mm Hg or 71–90 mm Hg with heart rate ≥ 108 beats per minute.
- Intervention (I): Pre-hospital plasma infusion (fresh frozen or lyophilized).
- Control (C): Standard-care resuscitation using crystalloid solution infusion.
- Outcome (O): Primary outcomes were all-cause mortality after 24 h, at 28–30 days post-injury, and the incidence of multiple organ failure. Secondary outcomes included the need for massive transfusion, vasopressor requirement within 24 h, INR on hospital arrival, 24-h total blood product volume (plasma, platelets, packed RBCs), and safety outcomes (incidence of adverse events, acute lung injury, transfusion reactions, and sepsis).

Exclusions comprised (1) in vitro and animal studies, (2) non-randomized trials (3), crossover trials, (4) observational studies, and (5) conference abstracts and posters.

### Study selection

Two authors (M.T. and A.S.) screened the titles and abstracts of retrieved records for eligibility after duplicate removal using the Covidence online tool. Full texts of the chosen studies were obtained and screened to determine their eligibility for meta-analysis. Any discrepancy was solved via discussion.

### Data extraction

Two reviewers (M.A. and B.A.) drafted and pilot-tested a data extraction sheet for study characteristics (including country, study design, total participants, main inclusion criteria, the primary endpoint, and follow-up duration), baseline characteristics (such as age, sex, group sizes, prehospital transport time and volume of crystalloid solution, initial Glasgow Coma Scale, comorbidities, injury severity score, abbreviated injury scale score for the head, and trauma type), and efficacy outcome data (involving mortality at 24 h and 1 month, acute lung injury, multi-organ failure, ventilator-free days, INR on admission, need for vasopressors within 24 h, massive transfusion, total 24-h volume of packed red blood cells, fresh frozen plasma, and platelets, and any adverse events). Reviewers (M.T. and A.S.) independently extracted this data, with disagreements resolved by involving M.A. to reach a consensus.

### Risk of bias and quality assessment

We employed the revised Cochrane collaboration tool for assessing the risk of bias in randomized controlled trials (ROB 2) [15]. Two reviewers (M.T. and A.S.) independently assessed the selected studies for risks related to selection, performance, reporting, attrition, and overall biases. In cases of disagreement, a consensus was reached involving a third one (M.A.). To evaluate the quality of evidence, two reviewers (M.A. and B.A.) followed the Grading of Recommendations Assessment, Development, and Evaluation (GRADE) guidelines [16, 17]. The quality of evidence for each outcome was presented, and any disagreements were resolved through discussion.

### Statistical analysis

Statistical analysis was performed via RevMan v5.3 software [18]. We utilized the pooled risk ratio (RR) for dichotomous outcomes with a 95% confidence interval (CI) and the mean difference (MD) with a 95% CI for continuous outcomes, or the standardized mean difference (SMD) for data with different units of measurement. Heterogeneity was assessed using the *I*-square and Chi-square tests. The Chi-square test identified the presence of heterogeneity, while the *I*-square test determined its extent. Significance for the Chi-square test was considered at an alpha level below 0.1, following guidelines in the Cochrane Handbook (chapter nine) (14), interpreting the *I*-square test: 0–40% (not significant), 30–60% (moderate heterogeneity), 50–90% (substantial heterogeneity). The analysis employed a fixed-effects model. We also conducted a meta-regression analysis based on the study-level covariate (transport time) using OpenMetaAnalyst.

## Results

### Search results and study selection

The search process involved screening and evaluating 1332 studies based on their titles and abstracts. After removing duplicates (625) and irrelevant studies (697), nine articles proceeded to full-text screening. Eventually, we included three RCTs [9, 10, 12] (Fig. S1).

### Characteristics of included studies

Three randomized clinical trials involving a total of 760 adults at risk of hemorrhagic shock due to trauma were included in our analysis, comprising 577 men and 184 women [9, 10, 12]. Two trials were carried out in the USA, while one was conducted in France. Comprehensive details and baseline characteristics of these studies can be found in Tables 1 and 2.

**Table 1:** Summary characteristics of the included RCTs

Study ID	Study design	Trial registry	Country	*N* of participants in each group, *N*. (%)		Type of plasma	Amount of plasma	Main inclusion criteria	Primary outcome	Follow-up duration
				Plasma	Control					
Jost et al. 2022	Multicenter, phase III RCT	NCT02736812	France	68 (50.7)	66 (49.3)	Lyophilized plasma	800 ml	Adult with a hemorrhagic shock of traumatic origin with SBP < 70 mmHg or Shock Index > 1.1	INR value at hospital admission	28-day follow-up
Moore et al. 2018	Single-center, phase II RCT	NCT01838863	USA	65 (52)	60 (48)	Fresh frozen plasma	500 ml	Injured adults with SBP 70 mm Hg or lower or 71–90 mm Hg and heart rate > 108 beats per min	Mortality within 28 days after injury	28-day follow-up
Sperry et al. 2018	Multicenter, cluster-randomized, phase III RCT	NCT01818427	USA	230 (45.9)	271 (54.1)	Fresh frozen plasma	500 ml	Injured adults with SBP < 90 mm Hg and heart rate > 108 beats per minute or if SBP < 70 mm Hg	Mortality within 30 days after injury	30-day follow-up

**Table 2:** Baseline characteristics of the participants

Study ID	Age (years) mean (SD)		Gender (male) *N* (%)		Prehospital transport time, mean (SD)		Prehospital volume of crystalloid solution, mean (SD)		Initial Glasgow Coma Scale score < 8, *N*. (%)			Injury severity score, mean (SD)		Abbreviated injury scale score for head, mean (SD)		Comorbidities, *N*. (%)		Trauma type, *N*. (%)			
	Plasma	Control	Plasma	Control	Plasma	Control	Plasma	Control	Plasma	Control		Plasma	Control	Plasma	Control	Plasma	Control	Blunt		Penetrating	
																		Plasma	Control	Plasma	Control
Jost et al. 2022	37.63 (17.19)	35.45 (16.98)	59 (86.8)	51 (77.3)	61.33 (16.66)	62 (18.95)	725 (397)	1017 (492.6)	10 (14.7)	11 (16.7)		29.67 (27.27)	25 (24.25)	N/A	N/A	12 (17.6)	6 (9.1)	40 (58.8)	40 (60.6)	28 (41.2)	26 (39.4)
Moore et al. 2018	36.33 (19.71)	33.33 (12.53)	52 (80)	51 (85)	19.33 (5.31)	17.33 (6.08)	150 (227)	283.3 (303.3)	N/A	N/A		26 (23.50)	24.83 (18.61)	0.67 (1.52)	0.83 (1.9)	10 (15.4)	8 (13.3)	30 (46.2)	32 (53.3)	35 (53.8)	28 (46.6)
Sperry et al. 2018	44.67 (20.89)	44.67 (23.85)	164 (71.3)	200 (73.8)	43 (14.17)	41.33 (13.42)	583 (932)	800 (1118)	103 (44.8)	129 (47.6)		23 (14.17)	20.67 (12.67)	1.67 (2.24)	1.33 (2.24)	N/A	N/A	187 (81.3)	226 (83.4)	46 (20)	49 (18.1)

### Risk of bias and quality of evidence

**Fig. 1:**
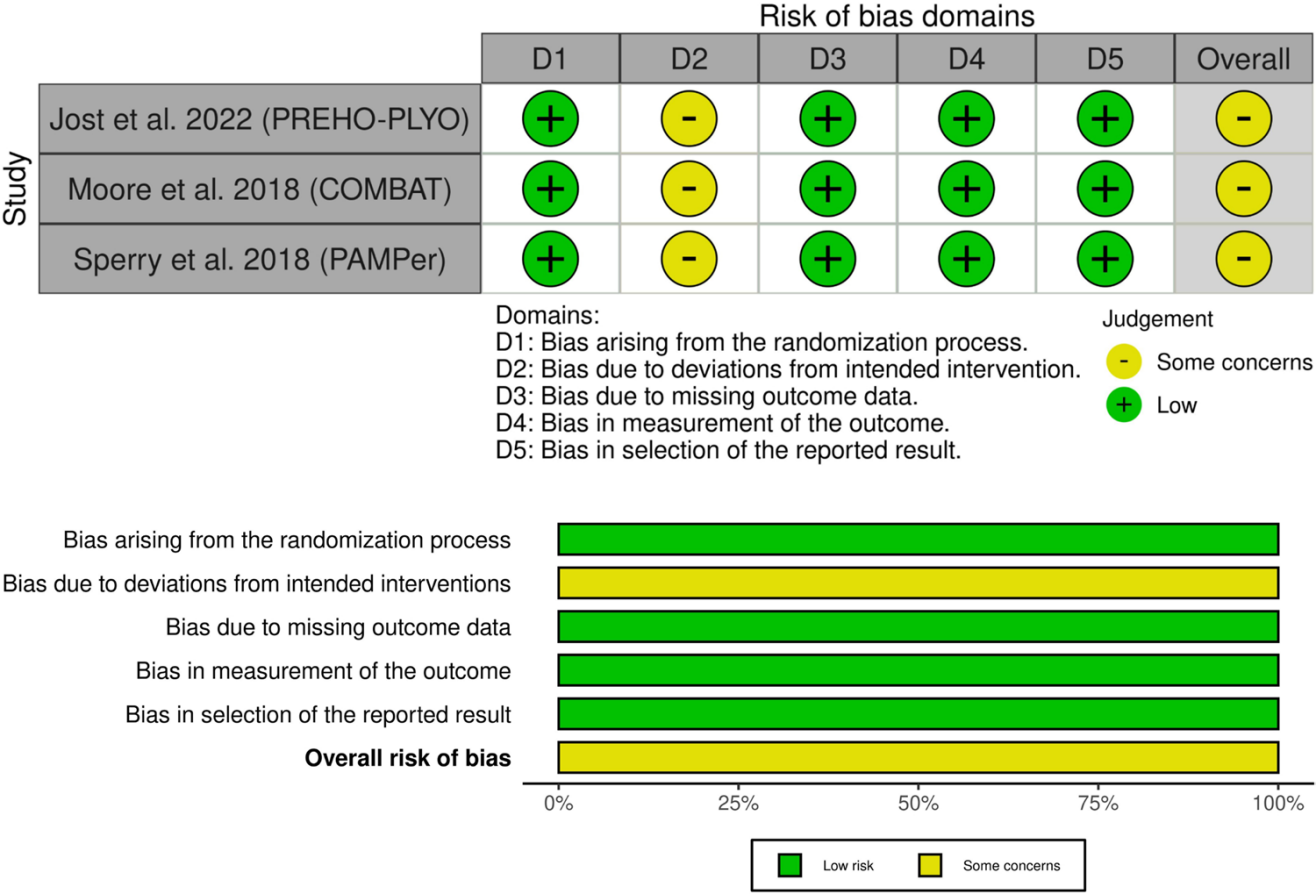
Quality assessment of the risk of bias in the included trials. The upper panel presents a schematic representation of risks (low = red, unclear = yellow, and high = red) for specific types of biases of each of the studies in the review. The lower panel presents risks (low = red, unclear = yellow, and high = red) for the subtypes of biases of the combination of studies included in this review

All three studies exhibited some concerns regarding the risk of bias [9, 10, 12]. Although all three studies were open-label, we did not consider that as a high risk of bias due to the absence of any deviations from the intended intervention due to trial context. A comprehensive risk of bias assessment is provided in Fig. 1. The quality of evidence is summarized in a GRADE evidence profile (Table 3).

**Table 3:** GRADE evidence profile

Certainty assessment							Summary of findings				
Participants (studies) follow-up	Risk of bias	Inconsistency	Indirectness	Imprecision	Publication bias	Overall certainty of evidence	Study event rates (%)		Relative effect (95% CI)	Anticipated absolute effects	
							With [comparison]	With [intervention]		The risk with [comparison]	Risk difference with [intervention]
24 h mortality											
760 (3 RCTs)	Not serious	Not serious	Not serious	Very serious^a^	None	⨁⨁◯◯ Low	72/397 (18.1%)	49/363 (13.5%)	RR 0.76 (0.54 to 1.06)	181 per 1000	**44 fewer per 1000** (from 83 fewer to 11 more)
30 days mortality											
760 (3 RCTs)	Not serious	Not serious	Not serious	Very serious^a^	None	⨁⨁◯◯ Low	105/397 (26.4%)	76/363 (20.9%)	RR 0.81 (0.63 to 1.05)	264 per 1000	**50 fewer per 1000** (from 98 fewer to 13 more)
Multiple organ failure											
760 (3 RCTs)	Not serious	Not serious	Not serious	Serious^b^	None	⨁⨁⨁◯ Moderate	160/397 (40.3%)	150/363 (41.3%)	RR 1.10 (0.95 to 1.27)	403 per 1000	**40 more per 1000** (from 20 fewer to 109 more)
Total 24-h volume of PRBCs units											
760 (3 RCTs)	Not serious	Not serious	Not serious	Very serious^b^	None	⨁⨁◯◯ Low	397	363	-	The mean management outcomes—total 24-h units of packed RBCS was 0	MD 0.83 lower (1.6 lower to 0.07 lower)
INR on admission											
259 (2 RCTs)	Not serious	Not serious	Not serious	Not serious	None	⨁⨁⨁⨁ High	126	133	-	The mean management outcomes—INR on admission was 0	MD 0.07 higher (0.02 higher to 0.12 higher)
Massive transfusion											
635 (2 RCTs)	Not serious	Not serious	Not serious	Very serious^a^	None	⨁⨁◯◯ Low	67/337 (19.9%)	48/298 (16.1%)	RR 0.83 (0.59 to 1.16)	199 per 1000	34 fewer per 1000 (from 82 fewer to 32 more)
Vasopressors needed within 24 h											
635 (2 RCTs)	Not serious	Not serious	Not serious	Not serious	None	⨁⨁⨁⨁ High	171/337 (50.7%)	138/298 (46.3%)	RR 0.91 (0.78 to 1.07)	507 per 1000	46 fewer per 1000 (from 112 fewer to 36 more)
Total 24-h volume of FFP units											
760 (3 RCTs)	Not serious	Not serious	Not serious	Very serious^b^	None	⨁⨁◯◯ Low	397	363	-	The mean management outcomes—total 24-h of packed FFP units was 0	MD 0.14 lower (0.53 lower to 0.26 higher)
Total 24-h volume of platelets units											
760 (3 RCTs)	Not serious	Not serious	Not serious	Not serious	None	⨁⨁⨁⨁ High	397	363	-	The mean management outcomes—total 24-h volume of platelets units was 0	MD **0** (0.12 lower to 0.12 higher)
Any adverse event											
651 (2 RCTs)	Not serious	Not serious	Not serious	Very serious^a^	None	⨁⨁◯◯ Low	20/345 (5.8%)	22/306 (7.2%)	RR 1.12 (0.65 to 1.94)	58 per 1000	7 more per 1000 (from 20 fewer to 54 more)
Acute lung injury											
626 (2 RCTs)	Not serious	Not serious	Not serious	Very serious^a^	None	⨁⨁◯◯ Low	80/331 (24.2%)	76/295 (25.8%)	RR 1.02 (0.79 to 1.33)	242 per 1000	5 more per 1000 (from 51 fewer to 80 more)
Transfusion reaction											
651 (2 RCTs)	Not serious	Not serious	Not serious	Very serious^a^	None	⨁⨁◯◯ Low	0/345 (0.0%)	1/306 (0.3%)	RR 3.53 (0.14 to 86.30)	0 per 1000	0 fewer per 1000 (from 0 to 0 fewer)
Sepsis											
651 (2 RCTs)	Not serious	Not serious	Not serious	Very serious^a^	None	⨁⨁◯◯ Low	1/345 (0.3%)	2/306 (0.7%)	RR 1.60 (0.26 to 9.96)	3 per 1000	2 more per 1000 (from 2 fewer to 26 more)

Explanations

^a^Confidence interval does not exclude the risk of appreciable benefit/harm, and the number of events is less than 300 events

^b^Confidence interval does not exclude the risk of appreciable benefit/harm

### Primary outcomes

**Fig. 2:**
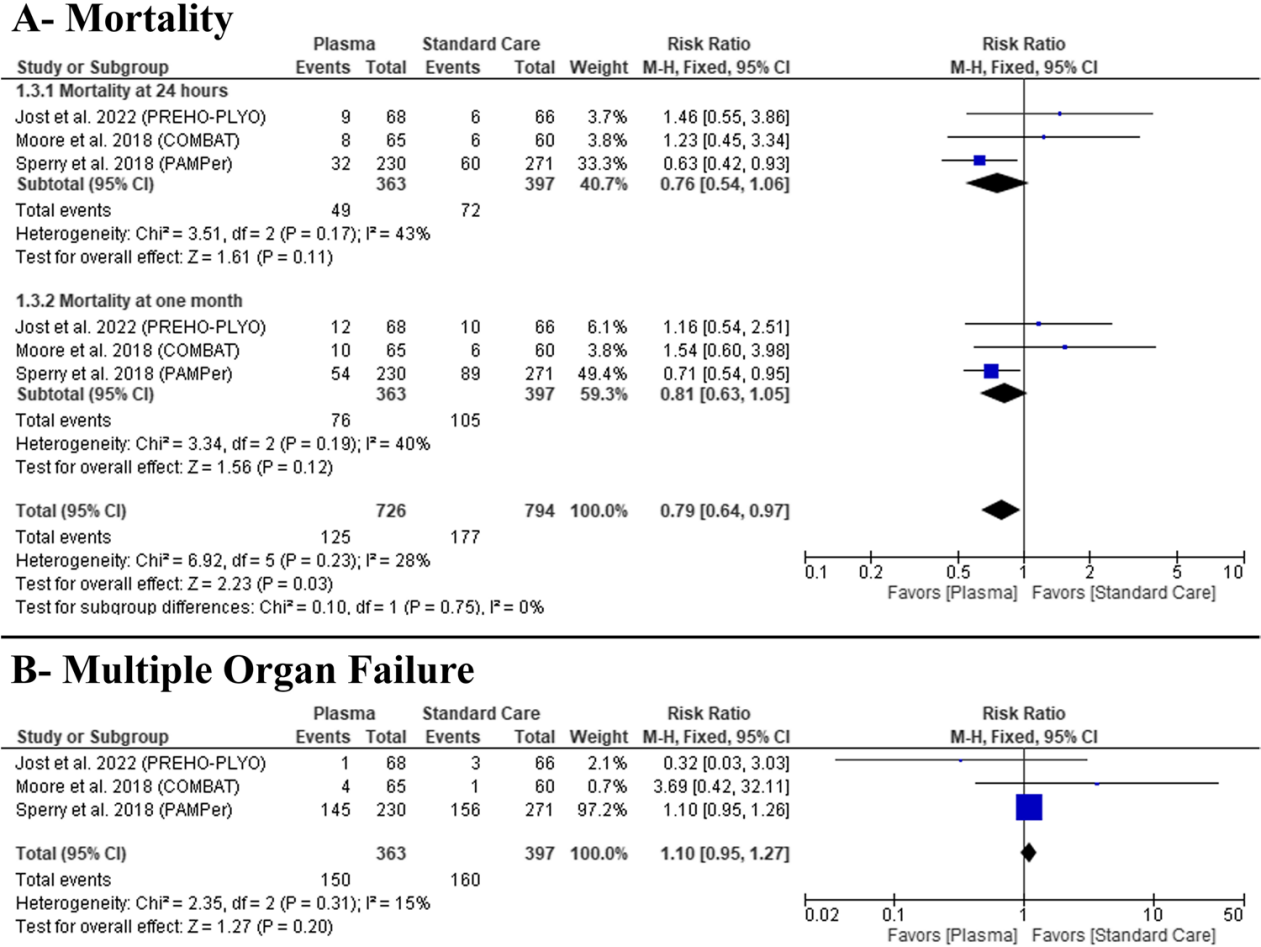
Forest plot of the efficacy outcomes (**A** mortality, **B** multiple organ failure), RR risk ratio, CI confidence interval

There was no difference between plasma infusion and standard care groups in 24-h mortality (RR: 0.76 with 95% CI [0.54, 1.06], *P* = 0.11) (low-quality evidence), 30 days mortality (RR: 0.81 with 95% CI [0.63, 1.05], *P* = 0.12) (low-quality evidence) (Fig. 2A, Table 3), and multiple organ failure incidences (RR: 1.1 with 95% CI [0.95, 1.27], *P* = 0.20) (moderate-quality evidence) (Fig. 2B, Table 3).

Pooled studies were homogenous in 24-h mortality (*P* = 0.17, *I*^2^ = 43%), 30-day mortality (*P* = 0.19, *I*^2^ = 40%), and multiple organ failure (*P* = 0.31, *I*^2^ = 15%).

Meta-regression analysis based on transport time showed no significant correlation between mortality at 24 h (*P* = 0.909) (Fig. S2) and mortality at 1 month (*P* = 0.789) (Fig. S3). However, meta-regression analysis showed that multiple organ failure was decreased with prolonged transport time (*P* = 0.127) (Fig. S4).

### Secondary outcomes

#### Management outcomes

The total 24-h volume of PRBC units was significantly decreased in the plasma group (MD: − 0.83 with 95% CI [− 1.60, − 0.07], *P* = 0.03) (low-quality evidence). Also, INR on arrival was significantly increased in the plasma group (MD: 0.07 with 95% CI [0.02, 0.12], *P* = 0.009) (high-quality evidence) (Fig. S5-A, Table 3). However, there was no difference between plasma infusion and standard of care groups in the total 24-h volume of packed FFP units (MD is − 0.14 with 95% CI [− 0.53, 0.26], *P* = 0.50) (low-quality evidence), total 24-h volume of platelets units (MD: 0.0 with 95% CI [− 0.12, 0.12], *P* = 1) (high-quality evidence) (Fig. S5-A, Table 3), massive transfusion (RR: 0.83 with 95% CI [0.59, 1.16], *P* = 0.27) (low-quality evidence), and vasopressor need during the first 24 h (RR: 0.91 with 95% CI [0.78, 1.07], *P* = 0.26) (high-quality evidence) (Fig. S5-B, Table 3).

Pooled studies were homogenous in total 24-h volume of PRBCs units (*P* = 0.19, *I*^2^ = 39%), INR on arrival (*P* = 0.37, *I*^2^ = 0%), total 24-h volume of packed FFP units (*P* = 0.42, *I*^2^ = 0%), total 24-h volume of platelets units (*P* = 1, *I*^2^ = 0%), massive transfusion (*P* = 0.21, *I*^2^ = 38%), and vasopressor need during the first 24 h (*P* = 0.55, *I*^2^ = 0%).

#### Safety outcomes

There was no difference between plasma infusion and standard care groups in the incidence of any adverse event (RR: 1.12 with a 95% CI [0.65, 1.94], *P* = 0.69) (low-quality evidence), acute lung injury (RR: 1.02 with a 95% CI [0.79, 1.33], *P* = 0.87) (low-quality evidence), transfusion reaction (RR: 3.53 with 95% CI [0.14, 86.30], *P* = 0.44) (low-quality evidence), and sepsis (RR: 1.60 with 95% CI [0.26, 9.96], *P* = 0.62) (low-quality evidence) (Fig. S6, Table 3).

Pooled studies were homogenous in the incidence of any adverse event (*P* = 0.40, *I*^2^ = 0%), acute lung injury (*P* = 0.29, *I*^2^ = 9%), and sepsis (*P* = 0.26, *I*^2^ = 21%).

## Discussion

Our investigation directly compared plasma infusion with standard-care resuscitation using crystalloid solutions in injured patients at high risk of hemorrhagic shock, evaluating key outcomes such as mortality, organ failure rates, and safety. The primary findings revealed no significant differences in 24-h mortality, 30-day mortality, or multiple organ failure between the two approaches. However, variations in secondary measures were noted, including the volume of PRBC units administered in 24 h and INR levels upon hospital arrival.

The lyophilized plasma, characterized by its freeze-drying process enabling room temperature storage, involves freezing the product and eliminating ice water from it [19]. Our examination combined studies using either fresh frozen or lyophilized plasma. Due to the limited number of available studies, a subgroup analysis to discern potential differences between these two types was not feasible. Nevertheless, previous studies directly comparing both types revealed no discernible differences [20].

Our primary investigation, comparing plasma infusion to standard-care resuscitation in high-risk hemorrhagic shock patients, yielded no significant differences in 24-h mortality, 30-day mortality, or multiple organ failure. Aligning with our findings, the utilization of prehospital plasma in the COMBAT trial did not demonstrate survival advantages for rapid urban trauma transit to level-1 centers [9]. Consistent results were reported with lyophilized plasma in the PREHO-PLYO trial [12]. In contrast, the PAMPer study showed a significant 9.8% decrease in 30-day mortality for severely injured trauma patients transported by air ambulance [10]. A secondary analysis of PAMPer revealed even lower mortality in patients with traumatic brain injury compared to the overall cohort [21]. Canton’s 2021 study presented divergent results, indicating decreased 30-day fatality rates and reduced lactate levels with prehospital plasma administration, proposing a potential dose–response relationship [22].

Reitz et al. (2020) demonstrated a survival benefit of prehospital plasma primarily observed in patients with blunt trauma but not in those with penetrating injuries. Moreover, no plasma-related adverse effects were demonstrated for penetrating injuries, highlighting the complexities surrounding mortality outcomes in trauma care and emphasizing the importance of considering specific patient characteristics and treatment modalities [20].

Furthermore, the post hoc analysis of the PAMPer and COMBAT trials introduced the concept of the 20th minute after transfer as a critical moment that could enhance survival rates at 28 days through prehospital plasma administration [19]. Notably, the PREHO-PLYO trial, requiring a minimum transfer time of 30 min, did not observe a similar effect [12]. Intriguingly, our meta-regression analysis yielded unexpected results, revealing no correlation between transport time and either 24-h or 1-month mortality. However, a noteworthy finding from our analysis was a decrease in multiple organ failure associated with prolonged transport time. This discrepancy underscores the critical role of timely intervention in the potential benefits of prehospital plasma administration, suggesting that factors like transfer duration might influence outcomes.

Observing a notable increase in INR levels upon patients’ arrival at the hospital aligns with outcomes documented in the COMBAT and PREHO-PLYO studies [9, 12]. It is noteworthy that the findings from the COMBAT trial did not reach statistical significance [9]. Despite the absence of reported INR outcomes in the PAMPer trial, a discernible pattern emerged where the plasma group received fewer blood components and displayed a lower prothrombin-time ratio compared to the standard-care group [10].

The seeming paradox of these results can be rationalized by acknowledging that plasma infusion does not consistently bring INR levels to within the normal range. Moreover, plasma demonstrates limited efficacy in correcting minimally elevated INR levels, as evident in two of our incorporated trials [9, 12]. This discrepancy may be elucidated by the fact that plasma units themselves can have elevated INR levels, reaching as high as 1.3 [23].

Our analysis further revealed a significant reduction in packed red blood cell units used over 24 h. Although this finding is supported by only one of the included trials, the large cohort size in this particular trial, in comparison to the others, significantly reinforces the credibility of this result [10].

In a broader context, the safety profile of plasma infusion was favorable, with no discernible significant differences when compared to standard care in terms of adverse events, transfusion reactions, sepsis, and multi-organ failure.

### Strengths and limitations

Our investigation has notable strengths. First, it is based on a systematic review and meta-analysis, ensuring a thorough evaluation of the evidence. We conducted a meticulous literature search, adhered to predefined inclusion criteria, and assessed the quality of included studies, enhancing the analysis’s reliability. Second, we included randomized controlled trials, considered the gold standard for evaluating interventions, bolstering the study’s credibility. Third, we examined outcomes like mortality, organ failure, blood product use, and coagulation parameters, offering a comprehensive view of plasma infusion’s impact on trauma patients at risk of hemorrhagic shock.

However, our study has limitations. To begin with, the included studies were small in number, limiting the statistical power. Future studies should consider larger sample sizes for more precise and generalizable results. In addition, there was significant heterogeneity in study populations, plasma products, and resuscitation protocols among the trials, potentially impacting our findings. Standardizing these parameters in future studies could reduce confounding effects. Furthermore, the amalgamation of studies utilizing both fresh frozen and lyophilized plasma, driven by the limited number of available studies, precluded a subgroup analysis based on the type of plasma used. Finally, our meta-regression analysis based on transport time only included three studies; therefore, it is not reliable for a definitive conclusion, and caution is required for its interpretation.

### Clinical and practical implications

Our study has important clinical implications. While we did not find significant differences in mortality and multiple organ failure, the reduced PRBC transfusion volume in the plasma group suggests potential benefits in managing trauma patients at risk of hemorrhagic shock by reducing transfusion-related complications.

Additionally, the increased INR in the plasma group upon hospital arrival highlights the importance of monitoring coagulation parameters and adjusting treatments accordingly. Future studies should also assess long-term patient outcomes and the cost-effectiveness of plasma infusion versus standard resuscitation.

## Conclusion

Our systematic review and meta-analysis suggest that plasma infusion does not significantly affect mortality and the incidence of multiple organ failure in trauma patients at risk of hemorrhagic shock. However, plasma infusion may be associated with a decrease in the total volume of packed red blood cell transfusions and an increase in INR on arrival at the hospital. These findings highlight the need for further research and well-designed trials to confirm the results and assess the clinical implications of plasma infusion in trauma patient management.

## Supplementary Information

Below is the link to the electronic supplementary material.

Supplementary file1 (DOCX 997 kb)
