# Supplementary material for: The efficacy and safety of pre-hospital plasma in patients at risk for hemorrhagic shock: an updated systematic review and meta-analysis of randomized controlled trials
Source: Eur J Trauma Emerg Surg. 2024 Feb 17;50(6):2697–707. doi: 10.1007/s00068-024-02461-7 (PMC11666795; doi:10.1007/s00068-024-02461-7)

**Title.**

**The efficacy and safety of pre-hospital plasma in patients at risk for hemorrhagic shock: an updated systematic review and meta-analysis of randomized controlled trials.**

**Running Title.**

**Pre-hospital plasma for hemorrhagic shock.**

**Authors.**

Mohamed Abuelazm^1^, Hazem Rezq^2^, Abdelrahman Mahmoud^3^, Mohammad Tanashat^4^, Abdelrahman Salah^5^, Othman Saleh^6^, Samah Morsi^7^, Basel Abdelazeem^8^.

**Affiliations.**

1. Faculty of Medicine, Tanta University, Tanta, Egypt.
2. Faculty of Medicine, Al-Azhar University, Cairo, Egypt.
3. Faculty of Medicine, Minia University, Minia, Egypt.
4. Faculty of Medicine, Yarmouk University, Irbid, Jordan.
5. Faculty of Medicine, Zagazig University, Zagazig, Egypt.
6. Faculty of Medicine, The Hashemite University, Zarqa, Jordan
7. Department of Radiation Oncology, UT Texas MD Anderson, Houston, Texas, USA.
8. Department of Cardiology, West Virginia University, Morgantown, West Virginia, USA.

**Keywords.**

Plasma; trauma; shock; bleeding; systematic review; meta-analysis.

**Corresponding Author:** Hazem Rezq

**E-mail:** HazemRezq.2020@azhar.edu.eg

| Database | Search Terms | Search Field | Search Results |
| --- | --- | --- | --- |
| PubMed | ("Prehospital plasma" OR "Fresh frozen plasma" OR "Lyophilized plasma") AND ("Hemorrhagic shock" OR Hypovolemia OR Hypoperfusion) | All Field | 270 |
| Cochrane | #1 (Prehospital plasma):ti,ab,kw OR (Fresh frozen plasma):ti,ab,kw OR (Lyophilized plasma):ti,ab,kw 1088  #2 (Hemorrhagic shock):ti,ab,kw OR (Hypovolemia):ti,ab,kw OR (Hypoperfusion):ti,ab,kw 1997  #3 #1 AND #2 50 | All Field | 50 |
| WOS | ("Prehospital plasma" OR "Fresh frozen plasma" OR "Lyophilized plasma") AND ("Hemorrhagic shock" OR Hypovolemia OR Hypoperfusion) | All Field | 390 |
| SCOPUS | TITLE-ABS ( ( "Prehospital plasma" OR "Fresh frozen plasma" OR "Lyophilized plasma" ) AND ( "Hemorrhagic shock" OR hypovolemia OR hypoperfusion ) ) | Title, Abstract | 249 |
| EMBASE | #3. #1 AND #2 373  #2. 'hemorrhagic shock':ti,ab,kw OR 39,900  hypovolemia:ti,ab,kw OR hypoperfusion:ti,ab,kw  #1. 'prehospital plasma':ti,ab,kw OR 'fresh frozen 11,960  plasma':ti,ab,kw OR 'lyophilized plasma':ti,ab,kw | All Field | 373 |

Table S1: Search terms and results in different databases.

Figure S1: PRISMA flow chart of the screening process.


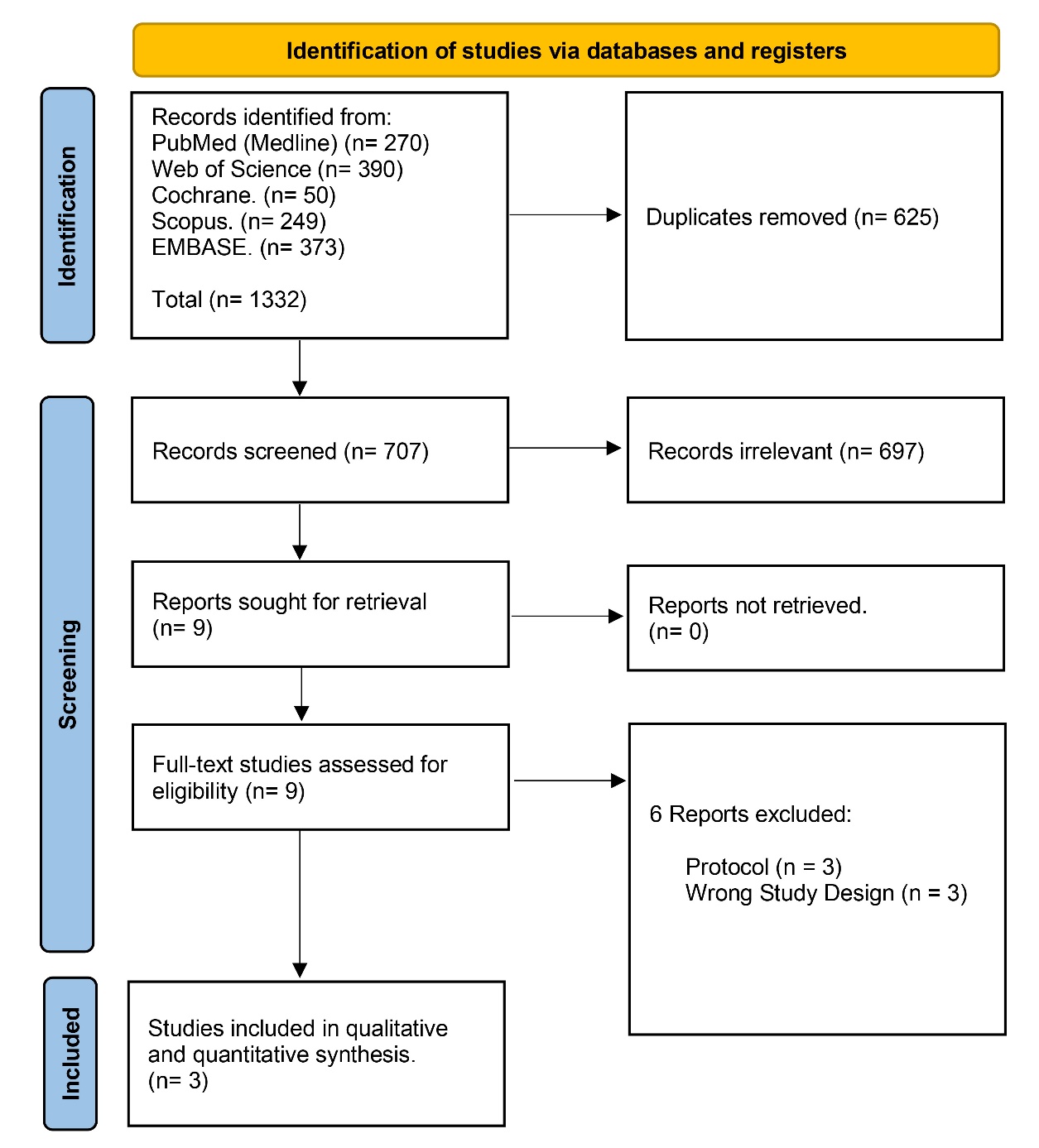


Figure S2: 24-h mortality.


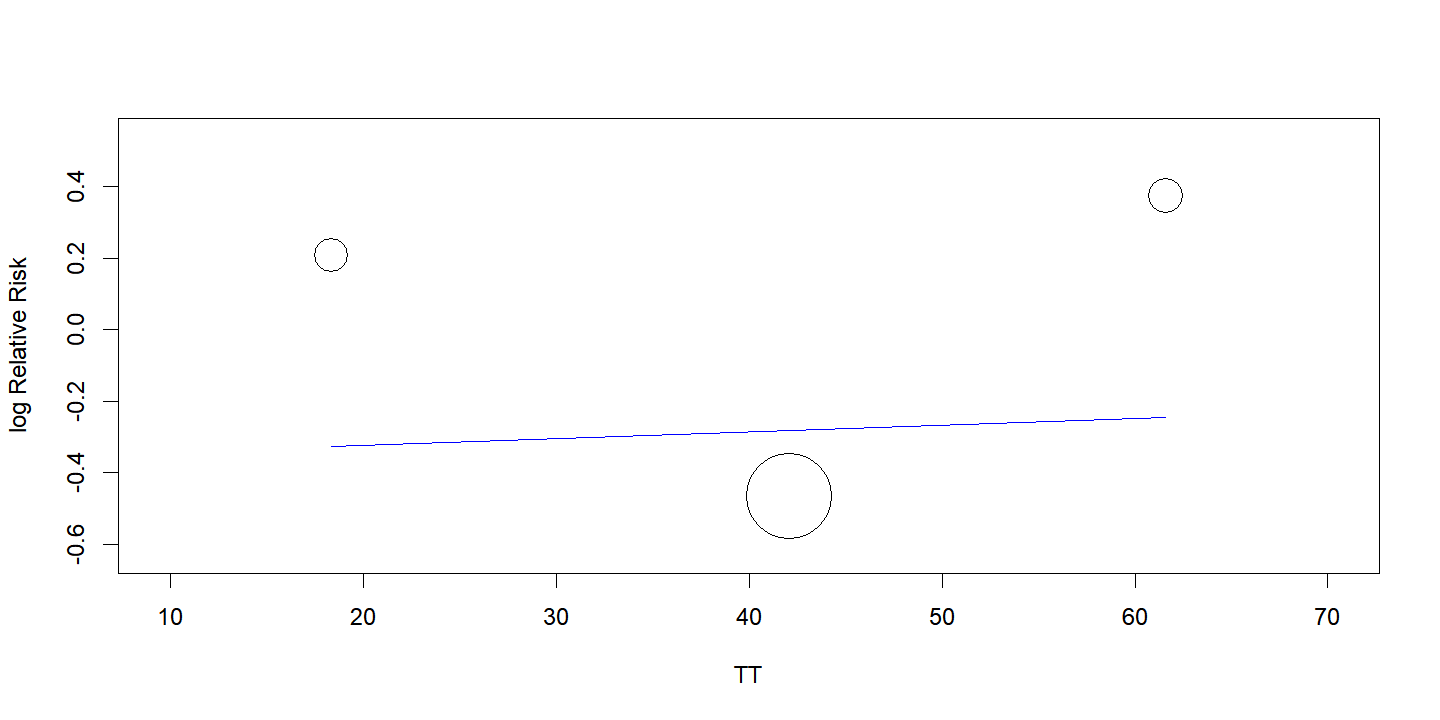


Figure S3: 1-month mortality.

**
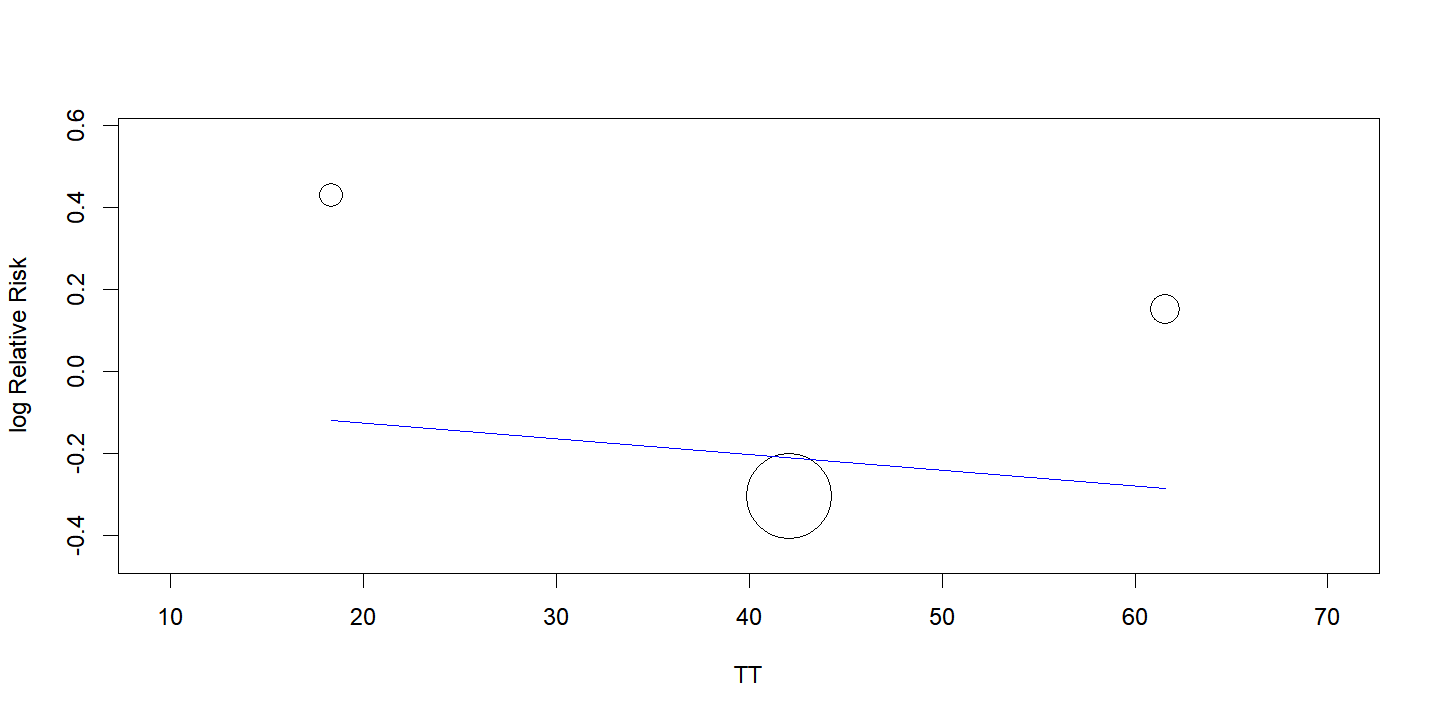
**

Figure S4: Multiple organ failure.

**
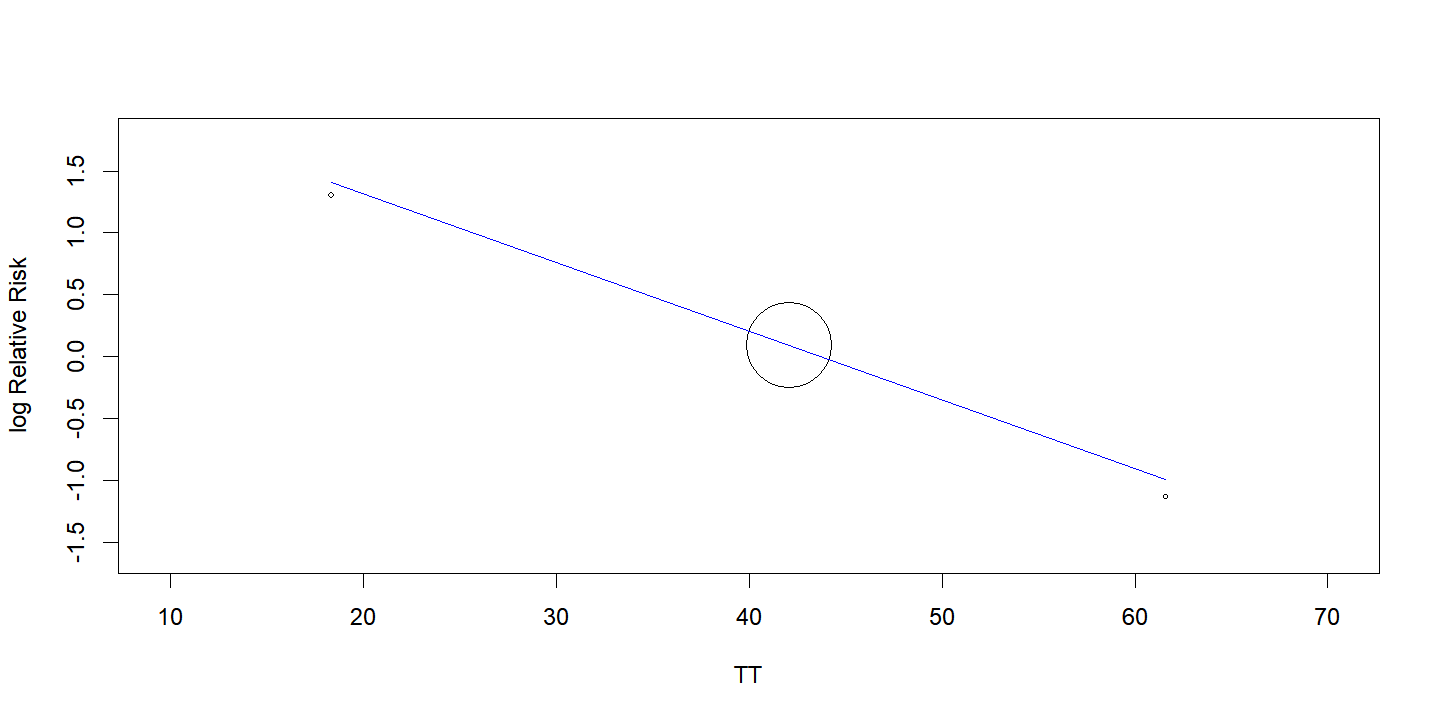
**

Figure S5: Forest plot of the efficacy outcomes (A1- Total 24-h units of packed RBCs, A2- Total 24-h of packed FFP units, A3- Total 24-h volume of platelets units, A4- INR on Admission, B1- Massive Transfusion, B2- Vasopressors needed within 24 hours), RR: risk ratio, CI: confidence interval; FFP: fresh frozen plasma, INR: international normalized ratio.


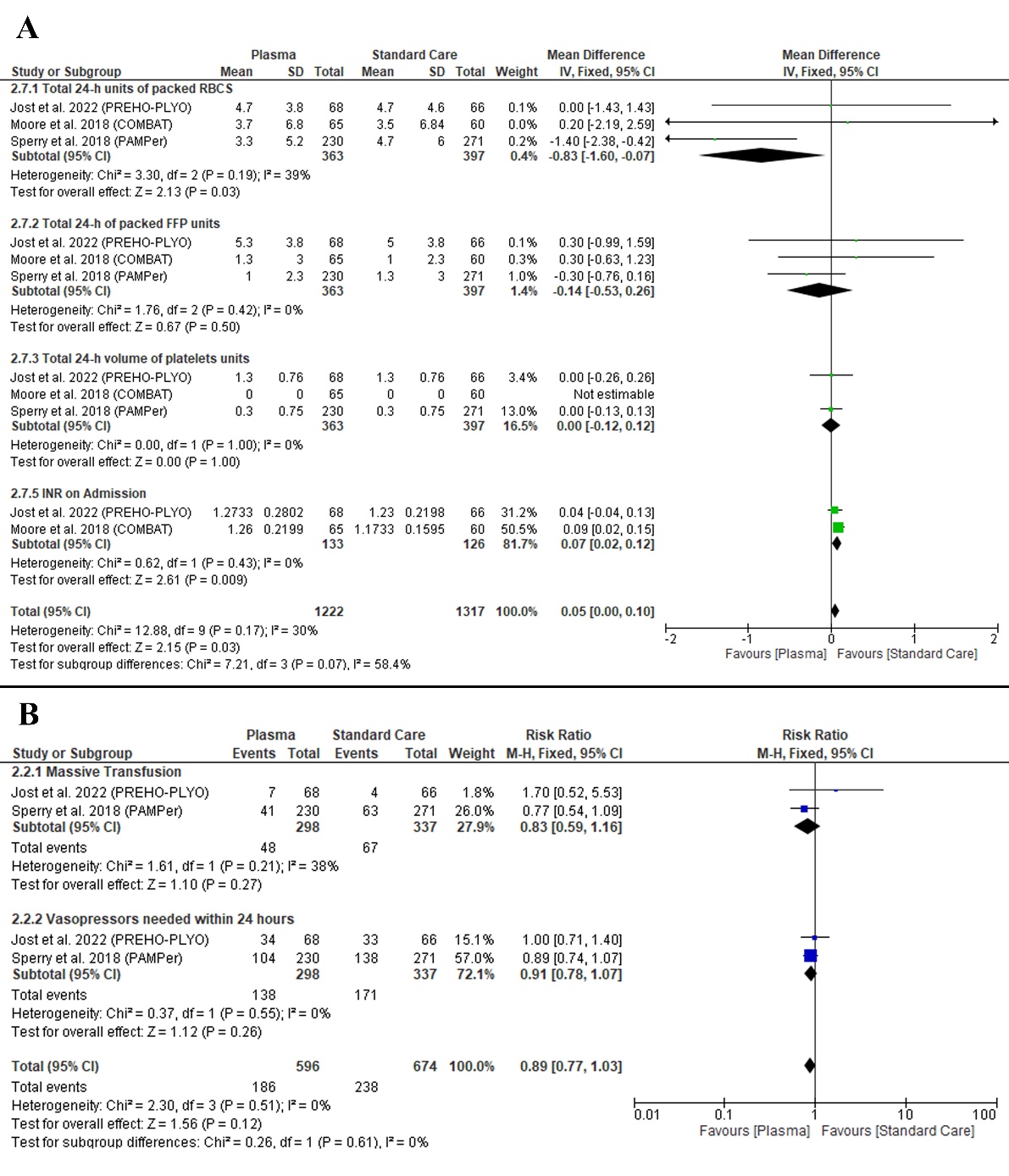


Figure S6: Forest plot of the safety outcomes (1- Any adverse event, 2- Transfusion reaction, 3- Sepsis, 4- Acute lung injury), RR: risk ratio, CI: confidence interval.


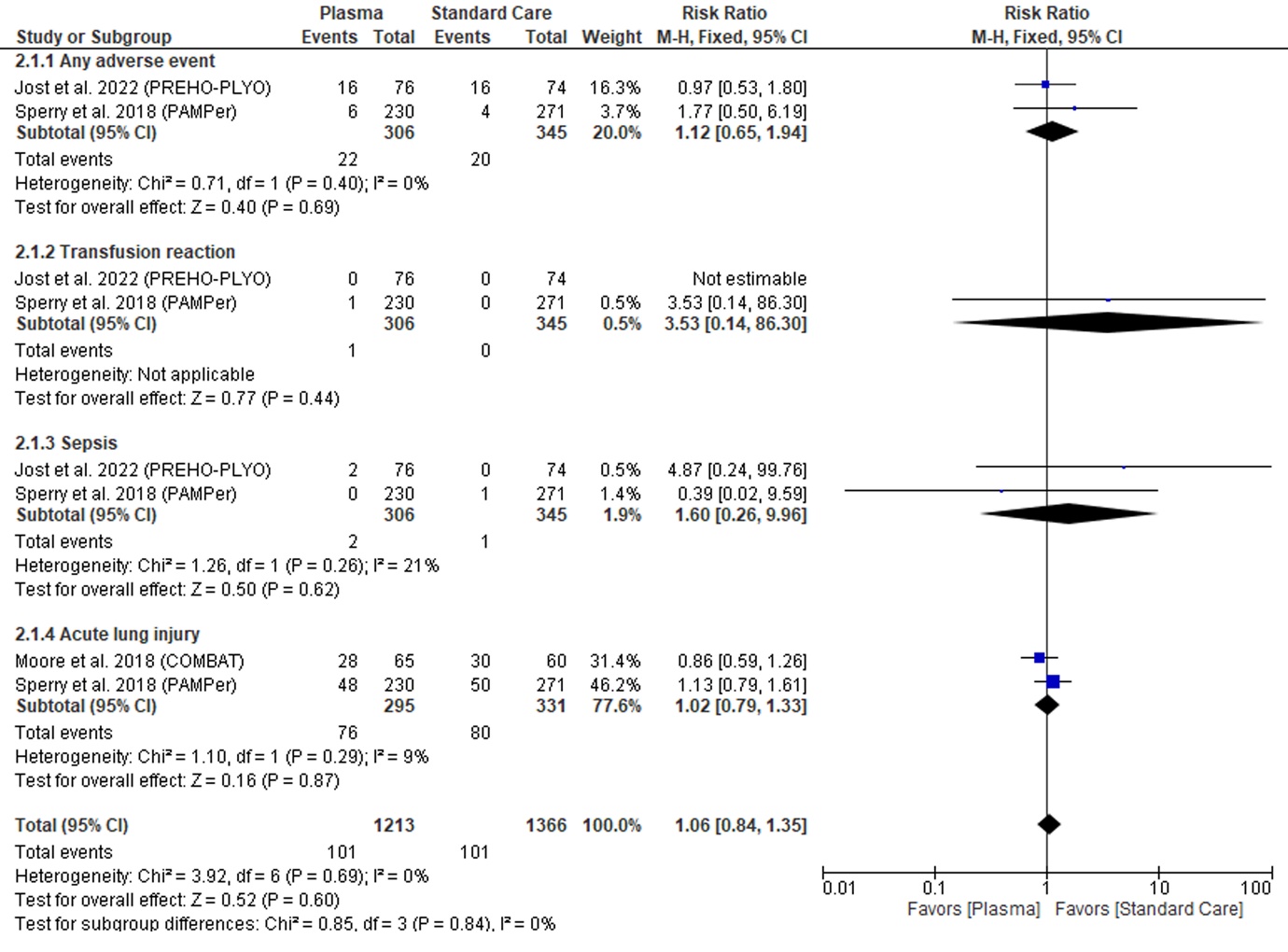

Supplement: Supplementary file 1 — Supplementary file1 (DOCX 997 kb) [file 68_2024_2461_MOESM1_ESM.docx]
